# Supplementary material for: The Triplex-Centric Assembly and Maturation of the Herpesvirus Procapsid
Source: Viruses. 2025 Aug 22;17(9):1153. doi: 10.3390/v17091153 (PMC12474303; doi:10.3390/v17091153)
Supplement: Supplementary file 1 [file viruses-17-01153-s001.zip › Heymann_supplement.pdf]

The triplex-centric assembly of the herpesvirus procapsid and its implications for capsid maturation

J Bernard Heymann

National Cryo-EM Program, Cancer Research Technology Program, Frederick National Laboratory for Cancer Research, Leidos Biomedical Research, Inc., Frederick, MD 21701, USA

Corresponding author:
jbheymann@me.com

Supplement

Alternative arrangements of triplexes

If we adhere to the rule that no MCPs attached to Tri1 subunits can interact, only four triplex orientation patterns are possible, the three alternates are shown in Figure S1. These occur in pairs, with the native one and the first alternate (Figure S1A) differing only in the rotation of Td. The other two alternates (Figure S1B,C) differ only in the rotation of Tb. The main difference between the pairs are in the rotations of Ta and Tc.

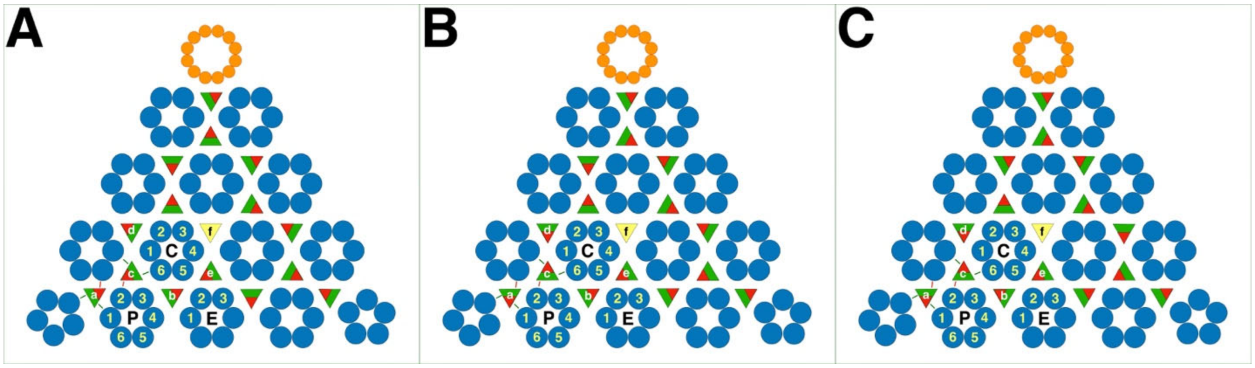

Figure S1: Alternative configurations of the triplexes adhering to the rule of excluding one unfavored arrangement of triplex pairs. Each orientation indicated by the red triangle corresponding to the position of Tri1. (A) Only Td is rotated compared to the correct configuration. (B) Rotation of Ta requires rotation of Tc. (C) The same configuration as C but with Tb rotated as well.

The arrangement of Tri1 subunits in the T=7dextro particle

The small particle composed of only MCP and Tri1 have orientations of the Tri1 subunits that resemble those of the full capsid (Figure S2).

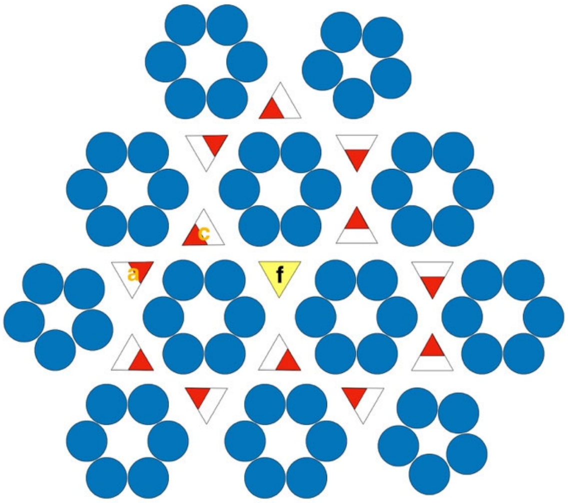

Figure S2: The T=7dextro particle composed of the MCP and Tri1 subunits (no scaffold). It appears that the Tri1 subunit orientations correspond to the Ta and Tc triplexes of the full capsid. This means that the Tri1 on the threefold may exhibit an unfavored arrangement. However, in the context of this particle and in the absence of Tri2 subunits, it may not be that meaningful. (Note: the dextro hand is assigned here as opposed to the laevo hand of the map shown in [1]. The reason is that their capsid reconstruction has the wrong hand [2] and it is assumed the same processing was done for both maps).

Comparison of maturation in HSV1 with the changes in HK97

The floor of HSV1 contains the Johnson fold of the bacteriophage HK97. Overlaying the structures of the two mature capsids show that the Johnson fold have a similar orientation, but the HK97 capsomers are more compact (Figure S3). Also apparent are the more elaborate HSV1 interfaces with neighboring capsomers involving the N-termini and dimerization domains (the parts sticking out in Figure S3).

The HK97 prohead II structure fits well within the envelope of the HSV1 procapsid (Figure S4). The skew in the hexamer of HK97 is much like the skew in the procapsid floor. As for HK97, the transformation of the procapsid during maturation is thought to be the rigid body rotation of the MCPs with remodeling of the intercapsomer interactions [3].

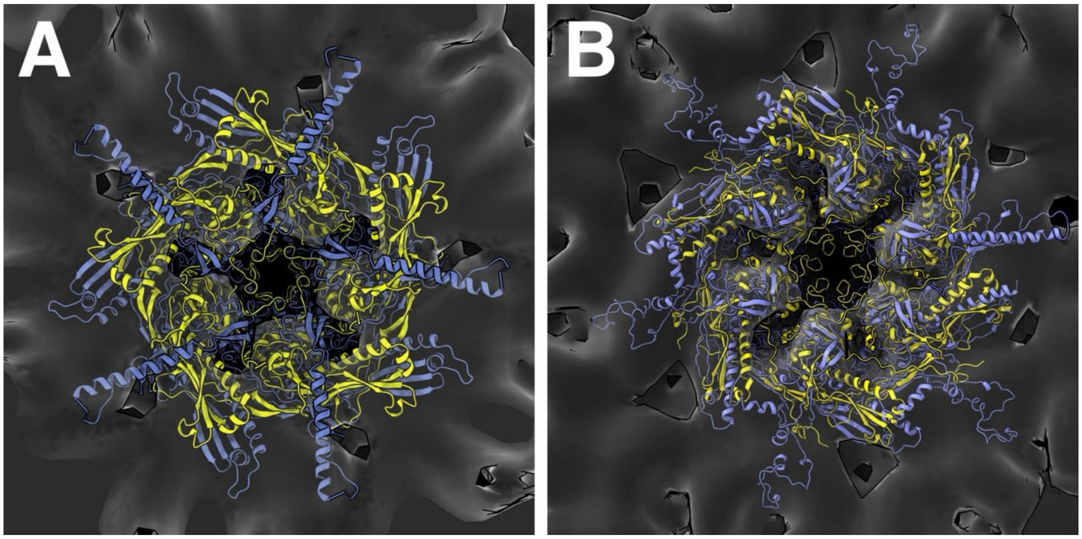

Figure S3: Comparison of the floor of HSV1 mature capsid (blue) with HK97 head II (yellow). (A) The HSV1 penton floor (blue) extends well beyond the HK97 pentamer (yellow). (B) The HSV1 P hexon floor also extends beyond the HK97 hexamer. The prominent HSV1 helix on the right connects to the penton. Map from [3], HSV1 structure from [4] (PDB 6CGR), and HK97 head II structure from [5] (PDB 2FT1).

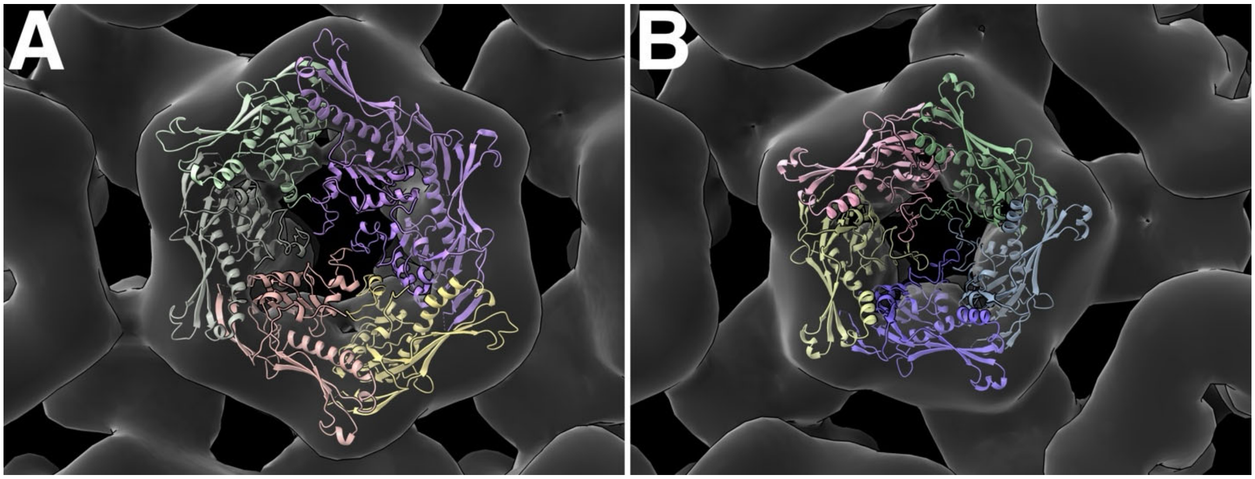

Figure S4: Comparison of the HK97 capsomers with the HSV1 procapsid map. (A) Hexamer fit into the P-hexon floor show approximately the same skew deviation from symmetry. (B) Pentamer fit into the penton. Map from [3], and HK97 prohead II structure from [6] (PDB 8CFA).

HSV1 capsid maturation movies

Movie: HSV1_capsid_maturation_2002.mov:
The linear arrangement of 17 maps reconstructed for the maturation of the HSV1 capsid was captured in a movie to highlight the changes observable around a resolution of ~20 Å [3]. The pertinent changes are the coalescing of the hexons, the closure of the capsid floor, and changes in the connections around the triplexes.

Movies: HSV1_pent_pd.mov, HSV1_phex_pd.mov, HSV1_ehex_pd.mov, HSV1_chex_pd.mov:

The 17 maps in the maturation series were expanding to 161 maps by interpolation. The upper turret domain of the HSV1 MCP solved by X-ray crystallography [7] was fitted into 161 maps and refined using a Monte Carlo Metropolis algorithm [8] implemented in the Bsoft program **bmonte** [9]. The movies show the Brownian-like motion of the upper domains as they coalesce into mature capsomers.

References

1. Saad, A.; Zhou, Z.H.; Jakana, J.; Chiu, W.; Rixon, F.J. Roles of triplex and scaffolding proteins in herpes simplex virus type 1 capsid formation suggested by structures of recombinant particles. *J. Virol.* **1999**, *73*, 6821-6830.
2. Cheng, N.; Trus, B.L.; Belnap, D.M.; Newcomb, W.W.; Brown, J.C.; Steven, A.C. Handedness of the herpes simplex virus capsid and procapsid. *J Virol* **2002**, *76*, 7855-7859.
3. Heymann, J.B.; Cheng, N.; Newcomb, W.W.; Trus, B.L.; Brown, J.C.; Steven, A.C. Dynamics of herpes simplex virus capsid maturation visualized by time-lapse cryo-electron microscopy. *Nat Struct Biol* **2003**, *10*, 334-341.
4. Dai, X.; Zhou, Z.H. Structure of the herpes simplex virus 1 capsid with associated tegument protein complexes. *Science* **2018**, *360*, doi:10.1126/science.aao7298.
5. Gan, L.; Speir, J.A.; Conway, J.F.; Lander, G.; Cheng, N.; Firek, B.A.; Hendrix, R.W.; Duda, R.L.; Liljas, L.; Johnson, J.E. Capsid conformational sampling in HK97 maturation visualized by X-ray crystallography and cryo-EM. *Structure* **2006**, *14*, 1655-1665, doi:S0969-2126(06)00392-3 [pii]
10.1016/j.str.2006.09.006.
6. Hawkins, D.E.D.P.; Bayfield, O.W.; Fung, H.K.H.; Grba, D.N.; Huet, A.; Conway, J.F.; Antson, A.A. Insights into a viral motor: the structure of the HK97 packaging termination assembly. *Nucl. Acids Res.* **2023**, *51*, 7025-7035, doi:10.1093/nar/gkad480.
7. Bowman, B.R.; Baker, M.L.; Rixon, F.J.; Chiu, W.; Quiocho, F.A. Structure of the herpesvirus major capsid protein. *EMBO J* **2003**, *22*, 757-765.
8. Heymann, J.B.; Trus, B.L.; Steven, A.C. Dynamics of the Protrusion Domain of Herpes Simplex Virus Capsid from Time-Resolved Cryo-EM and Molecular Modelling. *Microscopy and Microanalysis* **2005**, *11*, 1068-1069, doi:10.1017/S1431927605504148.
9. Heymann, J.B.; Belnap, D.M. Bsoft: Image processing and molecular modeling for electron microscopy. *J Struct Biol* **2007**, *157*, 3-18.
